# WIP-YAP/TAZ as A New Pro-Oncogenic Pathway in Glioma

**DOI:** 10.3390/cancers10060191

**Published:** 2018-06-09

**Authors:** Sergio Rivas, Inés M. Antón, Francisco Wandosell

**Affiliations:** 1 Centro Nacional de Biotecnología (CNB-CSIC), Darwin 3, 28049 Madrid, Spain; srivas@cnb.csic.es; 2 Centro Nacional de Biotecnología (CNB-CSIC), 28031 Madrid, Spain; 3 Centro de Biología Molecular Severo Ochoa (CSIC-UAM), Nicolás Cabrera 1, 28049 Madrid, Spain; 4 Centro de Investigación Biomédica en Red de Enfermedades Neurodegenerativas (CIBERNED), Valderrebollo 5, 28031 Madrid, Spain

**Keywords:** signalling in cancer, glioma, CSCs, TICs, proliferation, survival, YAP/TAZ, Akt, WIP

## Abstract

Wild-type p53 (wtp53) is described as a tumour suppressor gene, and mutations in p53 occur in many human cancers. Indeed, in high-grade malignant glioma, numerous molecular genetics studies have established central roles of RTK-PI3K-PTEN and ARF-MDM2-p53 INK4a-RB pathways in promoting oncogenic capacity. Deregulation of these signalling pathways, among others, drives changes in the glial/stem cell state and environment that permit autonomous growth. The initially transformed cell may undergo subsequent modifications, acquiring a more complete tumour-initiating phenotype responsible for disease advancement to stages that are more aggressive. We recently established that the oncogenic activity of mutant p53 (mtp53) is driven by the actin cytoskeleton-associated protein WIP (WASP-interacting protein), correlated with tumour growth, and more importantly that both proteins are responsible for the tumour-initiating cell phenotype. We reported that WIP knockdown in mtp53-expressing glioblastoma greatly reduced proliferation and growth capacity of cancer stem cell (CSC)-like cells and decreased CSC-like markers, such as hyaluronic acid receptor (CD44), prominin-1 (CD133), yes-associated protein (YAP) and transcriptional co-activator with PDZ-binding motif (TAZ). We thus propose a new CSC signalling pathway downstream of mtp53 in which Akt regulates WIP and controls YAP/TAZ stability. WIP drives a mechanism that stimulates growth signals, promoting YAP/TAZ and β-catenin stability in a Hippo-independent fashion, which allows cells to coordinate processes such as proliferation, stemness and invasiveness, which are key factors in cancer progression. Based on this multistep tumourigenic model, it is tantalizing to propose that WIP inhibitors may be applied as an effective anti-cancer therapy.

## 1. Role of Actin in Cell Migration and Proliferation

Tumour transformation involves not only genetic reprogramming but also a change in cell morphology associated with epithelial–mesenchymal transition (EMT). It is clear that the actin cytoskeleton contributes to several cellular properties that are altered in tumour cells, where the oncogenic programme boosts proliferation, migration and/or differential adhesion. Thus, the increase in migratory capacity, or possible lack of substrate adhesion (anchorage independence) and the capacity to colonize other tissues depend largely on the actin cytoskeleton [1,2]. Cellular migration and invasion require integration of several processes that include local modulation of the cytoskeleton, contractile forces, recycling of substrate-adhesion structures and, finally, generation of specialized domains that mediate focal degradation of the extracellular matrix (ECM). At a cytoskeletal level, actin filaments (known as F-actin or microfilaments), composed of actin and a plethora of actin-regulating proteins, play an essential role in physiological and pathological migration.

“Podosomes” and “invadopodia” are actin-rich protrusions that drive invasion in normal and cancer cells [3,4,5]. They are associated with secretion and/or activation of matrix metalloproteases (MMP) and the subsequent degradation of the ECM, allowing cell invasion which is key to many oncogenic transformation; for review see [6].

## 2. WIP Structure and Function

The proteins that make up podosomes and invadopodia include actin, the actin-related protein (Arp)2/3 complex, (neural)-Wiskott–Aldrich Syndrome protein (N-WASP) [7,8], and WASP-interacting protein (WIP), among others [6,9]. The central core of actin polymerization is the nucleating Arp2/3 complex and a group of proteins that regulates the polymerization. Indeed, WASP was identified as a member of a family of proteins involved in microfilament organization which includes N-WASP and Wiskott-Aldrich syndrome protein family member 1 (WAVE1/Scar) [7,10,11,12,13]. WASP homologues have been identified in many eukaryotes from yeast to mammals, playing a critical role in the linkage of Cdc42-activation signals to actin microfilaments. Almost all members of Rho family of GTPases, belonging to the Ras superfamily, have been shown to regulate intracellular actin dynamics, but only two elements have been associated with (N-)WASP. Indeed, several data indicated that Cdc42 and Rac, bind directly to a protein implicated in the immunodeficiency disorder Wiskott–Aldrich syndrome [14,15]. Though structurally and functionally very similar, WASP is expressed only in hematopoietic cells [1,16] whereas N-WASP is ubiquitously expressed [13]. Both can form complexes with proteins that interact with actin, and with other proteins that participate in the formation of podosomes or invadopodia such as cortactin, myosin II, Nck, and Tks5/FISH [17,18]. 

The human WIP protein (503 aa in length) is proline rich, showing high sequence similarity to the yeast protein verprolin [17,18,19], and 95% identity with murine WIP. Two additional members of the protein family have been described: corticosteroid responsive (CR16) and WIP-related/WIP CR16 homologous (WIRE/WICH) [20,21]. WIP is ubiquitously expressed, but at higher levels in lymphoid cells [17]. Many reports have indicated that WIP is a multifunctional protein [19]; however, details of many of its biological functions are far from being understood.

Different structural and functional motifs have been described in WIP [22,23]. WIP binds WASP via its C-terminus (aa 461–485), and could bind actin via a KLKK motif within its WH2 domain [22,24,25]. WIP also has three ABM2 (actin-based mobility 2) profilin-binding motifs, in addition binding the adapter proteins Nck [26] and Crk L [27].

The interaction of (N-)WASP and WIP is essential to many cellular functions; (N-)WASP functions are regulated by WIP, inhibiting actin nucleation in vitro by Arp2/3 mediated by the activation of (N-)WASP through the GTPase Cdc42 [8]. In the absence of WASP, cells do not form podosomes and their chemotactic responses are deficient [28]. Similarly, in dendritic cells (DC) derived from WIP-deficient mice (WIP−/−) [18], the stability and localization of WASP was compromised, and therefore the formation of podosomes, migration and degradation of the ECM was reduced [9,29]. Indeed, we reported that WIP contributes to both amoeboid and mesenchymal migration [29,30]. Data from ours and other groups support the participation of WIP in prototypical cellular activities such as proliferation, migration, EMT transition and differentiation, as well as a role in the regulation of actin polymerization essential for formation/function of invadopodia [18,19,31,32].

More recently, we demonstrated that WIP is not only highly expressed in some tumour types, but is also essential for maintenance of tumour subpopulations denoted as “cancer stem cells” (CSCs) or “tumour-initiating cells” (TICs) [33], a phenotype frequently responsible for advancement to more aggressive stages of disease, at least in some tumour types [34].

## 3. Tumour Proliferation and CSCs: Glioma as a Tumour Model

The expression of stem-like markers, such as hyaluronic acid receptor (CD44), prominin-1 (CD133), nestin, or sex-determining region Y (SRY)-box 2 (Sox2) in several tumour types led to proposal of the CSC/TIC hypothesis some years ago. These tumour subpopulations, either designated as CSC or TIC depending on authors, were operationally defined not only by the presence of stem-like makers but for some functional characteristics [33,34]. For instance, these stem-like tumour populations possess the ability to self-renew and produce differentiating progeny, much like their normal counterpart. The origin of this tumoural subpopulation is still a matter of debate, perhaps not generated from “normal” stem cells or from a “unique” cell type. They are most likely generated by anomalous combinations of mutations (oncogenic and tumour-suppressor mutations) that cause the stem-like phenotype and subsequent accumulation of mutations that boost oncogenic progression, maintaining self-renewal capabilities and proliferation. An updated framework for understanding the significant diversity of neoplastic diseases, with ten hallmark capabilities acquired by normal cells evolving to a neoplastic state, was proposed by Hanahan and Weinberg [35,36]. Among them, sustained cell survival and proliferation in combination with evading apoptotic checkpoints are early capabilities enhanced in initially transformed cells to form a tumour [35]. In this proposal, CSC proliferation plays a key role in the generation and maintenance of this oncogenic progression enhancing proliferative signalling, improving migratory capabilities, and evading tumour suppressor genes bypassing cell death checkpoints.

Glioblastoma (GB) is the most common and aggressive subtype of the malignant gliomas, and is characterized by intense proliferation, invasion, and intratumoural heterogeneity. Although the CSC hypothesis was initially supported in human leukaemia [37,38], similar populations of cells with stem-like properties have been described in many solid tumours (for a review see: [39]). Indeed, a CD133+ cell subpopulation isolated from brain tumours exhibited CSC properties in vitro and in vivo [40,41,42]. Additionally, in high-grade malignant gliomas, several molecular genetic studies have established mutations in diverse pathways promoting oncogenic capacity, such as RTK-PI3K-PTEN and ARF-MDM2-p53 INK4a-RB [43]. The working hypothesis is that deregulation of these signalling pathways permits autonomous growth, and these initially transformed cells may acquire a tumour-initiating “stemness” phenotype responsible for advancement to more aggressive stages of disease (World Health Organization grade I to grade IV) [44]. In grade IV gliomas, mutations in the gene coding for phosphatase and tensin homolog (PTEN) and in the gene of tumour suppressor protein p53 (TP53) are the most common.

Indeed, in tumour cells obtained from human glioma explants, we demonstrated that the majority (3 of 5) had previously-described mutations in p53, and most had the capacity for anchor-independent growth and expressed stem markers such as CD133, as reported for similar CSCs [45]. Though also present in gliomas grown in serum-rich medium, the CD133+ subpopulation was significantly increased after growth in stem-like defined medium. We confirmed that the mutant versions of p53 were essential to maintain the oncogenic phenotype, while reduction of p53 expression by specific shRNA strongly reduced proliferation of the gliomas assayed, with a clear correlation to the reduction of CD133+ cells [45].

## 4. WIP Controls YAP/TAZ Stability in Mutant p53 Gliomas

The aim of our work was to identify new participating proteins within the mechanisms through which mtp53 generates and maintains the CSC phenotype in glioma. In addition, several studies have described that mtp53 may act through several tyrosine kinase receptors (RTK) and integrin signalling [46,47,48,49], linking the mutation with several pro-oncogenic hallmarks [35].

The first data we observed was a significant, direct correlation between the proliferation capacity of numerous mtp53 gliomas and the presence of high levels of WIP [33,45]. This correlation was initially described in human samples [33], with Yes-associated protein (YAP) and transcriptional co-activator with PDZ-binding motif (TAZ) directly implicated in tumour development and CSCs [50]. Indeed, YAP and TAZ bind Axin, which allows the YAP/TAZ/β-catenin complex to interact with its ubiquitin ligase βTrCP, facilitating ubiquitination of the complex and its subsequent degradation [51,52]. This mechanism may coordinate the regulation of these co-transcription factors by degradation. Alternatively, the degradation complex with YAP/TAZ/β-catenin can be sequestered in multivesicular bodies (MVB) of the endosomal compartment, which blocks degradation at a key step in Wnt signalling induction [53].

As mentioned above, YAP/TAZ regulate several processes in development, tissue homeostasis and cancer [2,50,54]. In addition, some data indicated that YAP enhances the proliferative transcriptional activity of mutant p53 proteins [55]. It has also been reported that actin polymerization regulates this pathway, affecting YAP/TAZ-mediated transcription [56]. It is also known that WIP can promote nuclear transit of myocardin-related transcription factor-serum response factor (MRTF-SRF), which is regulated by actin polymerization [57]. As both transcription factors depend on actin polymerization to reach the nucleus, we considered the possibility that WIP regulates YAP/TAZ.

We showed that the reduction of YAP/TAZ mediated by WIP knockdown is not compensated by either Jasplakinolide (F-actin stabilizer) or by latrunculin A1 (F-actin-depolymerizing agent). This finding strongly supports that the WIP-mediated stability of YAP/TAZ is not dependent on actin polymerization [33].

The way actin controls YAP/TAZ distribution and whether the cellular effects of WIP expression are exclusively due to a consequent increase of YAP/TAZ remain open questions. The identification of nuclear N-WASP and WIP support the option of a secondary effect, possibly unrelated to actin.

## 5. Akt Plays an Essential Role in the mtp53/WIP Pathway in Gliomas

As previously mentioned, one of the most commonly-modified pathways in gliomas is PI3K-PTEN-Akt, which is activated in normal and cancer cells [58] and controls tumour cell proliferation [59,60] and/or maintenance of the tumour phenotype [61]. Akt was initially described as the human homolog of a viral oncogene [62,63], and belongs to the AGC family of serine/threonine kinases [64]. Akt is involved in many biological processes and pathologies, such as metabolism regulation, cell growth, survival, proliferation, cancer, and neurodegenerative disorders [58,65]. Three Akt isoforms have been described in mammals, sharing high homology at the protein level, and they are encoded by three different closely-related genes (Akt1, Akt2 and Akt3) [58,66,67]. Many extracellular signals induce Akt activation through PI3K, which is based on phosphorylation of two residues, threonine 308 and serine 473, by phosphoinositide-dependent kinase-1 (PDK1) and mammalian target of rapamycin complex 2 (mTORC2), respectively [68]. Additionally, integrin-linked kinase (ILK) has been described to activate Akt [69].

It is largely recognized that hyper-activation of Akt plays an important role in cancer [70,71,72,73,74]. Akt is frequently activated in human cancers (for a review see [58]), either directly by over-expression/mutation or indirectly through alterations to PTEN. This over-activation promotes protection against apoptosis and “proliferation enhancement”, among others, two major prerequisites for cancer progression [74,75]. However, the exact role of each Akt isoform in tumour development is not completely clear. For instance, Akt1 is essential for the propagation of breast cancer, whereas ablation of Akt2 inhibits apoptosis and delays tumour involution [76,77]. In contrast to Akt1, which accelerates the induction of mammary tumours in transgenic mice, Akt2 can promote metastasis of tumour cells without affecting the latency of tumour development in certain systems [78]. We recently analysed the role of Akt isoforms in survival and self-renewal of TICs, as well as the correlation between Akt activity and the CSC/EMT phenotype in breast cancer cells [33,79]. In different breast cancer models, Akt1 appears to play a fundamental role in the propagation of such tumours [70,71,72,73,74] by enhancing not only proliferation and survival, but also cell migration. An increase in mobility and loss of polarity is a general characteristic of many tumours, and it may determine their aggressiveness and metastatic potential [80,81]. Thus, hyper-activation of some elements downstream of Akt may aggravate the migratory/invasive capacity of tumour cells (for a review see [65]).

In an attempt to identify the signalling downstream of mtp53 that may link Akt-mediated survival and proliferation with WIP in glioma CSCs, we compared WIP levels with Akt activity. We found a good correlation between high WIP levels and the level of active Akt and Erks in human glioma samples. In addition, astrocytes transformed with mtp53 resulted in a remarkable activation of Akt and Erk [33,45]. In support of initial observations, anchor-independent growth was severely hampered after treating glioma CSCs with PI3K inhibitors, showing reduced WIP and nestin in clear correlation with low levels of Akt activity [33,45]. This prompted us to propose that Akt or other downstream elements are linked with the mtp53 oncogenic pathway.

## 6. Rac and PAK among Others Control the Pro-Oncogenic Signaling of WIP

To identify proteins that can modulate pro-oncogenic signalling of WIP, or may be just downstream elements, we used two complementary approaches, first using the WIP-tumoral capacity as a reporter and second, following the presence of nuclear YAP/TAZ.

Thus, we used specific shRNA or specific inhibitors to block tumour growth taking into account some of the data already published. For instance, signalling by Ras-related C3 botulinum toxin substrate 1 (Rac) and serine/threonine p21-activated kinase (PAK) is essential for ErbB2-mediated transformation of human breast epithelial cancer cells [82]. Besides PI3K, Rac and PAK were recently implicated in a newly-reported clathrin-independent system of endophilin-mediated endocytosis (FEME) of receptors [83]. Indeed, Akt may phosphorylate PAK at serine 21, modulating its interaction with Nck [84], a partner of the WIP-(N)-WASP complex. In our cellular model of YAP/TAZ stabilization mediated by WIP overexpression, we showed that either a Rac inhibitor (NSC23766), or an allosteric inhibitor of Pak1 (IPA-3), or formin inhibitor (SMIFH2), among others, reversed WIP’s ability to stabilize YAP/TAZ. In conclusion the stability of YAP/TAZ WIP-mediated required the activity of RAC, or PAK or formin.

**Scheme 1:**
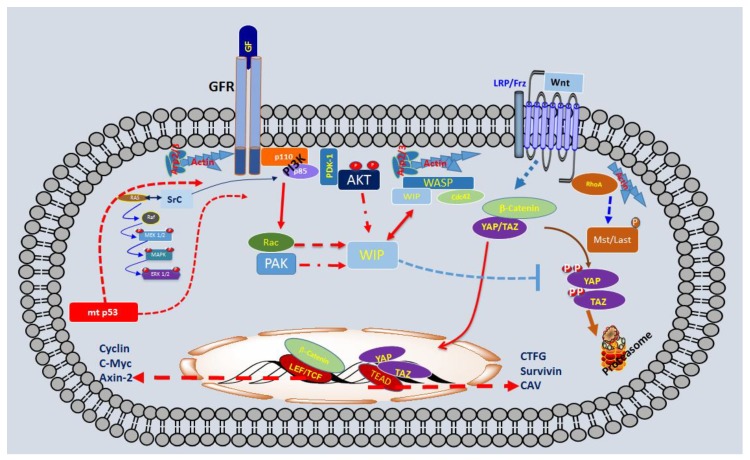
Schematic representation of some elements that play a key role in the Tumour-initiating cells (TICs) derived from gliomas.

Thus, we reported that Rac, PAK, and formin are essential for WIP to boost cell proliferation and promote tumours. This was supported by the observation that overexpression of Rac-V12 or constitutively active PAK (CA-PAK) or Protein diaphanous homolog 2 (mDia2) rescues the phenotype caused by the lack of WIP [33]. All these data strongly support the hypothesis that WIP exerts powerful control on receptor/integrin signalling to regulate YAP/TAZ and β-catenin stability through MVB dynamics, which may allow efficient sequestration of the APC/Axin/GSK3 destruction complex and promote YAP/TAZ/β-catenin stability. In a second step, this increase of YAP/TAZ and/or β-catenin would preserve the phenotype and enhanced proliferation of CSC/TICs. To summarize our working hypothesis, we have described some of the components of this new oncogenic pathway (Scheme 1). 

In conclusion, we have demonstrated that WIP drives a new mechanism that stimulates growth signals, promoting YAP/TAZ stability in a Hippo-independent fashion, which allows cells to coordinate key factors in cancer progression, such as proliferation, stemness and invasiveness. 

The lack of WIP impedes proliferation in mp53 positive gliomas, whereas the over-expression increases proliferation and CSC markers in astrocytes. In both cases, the presence/expression of YAP/TAZ is essential to maintain proliferation and stemness. With all these data we propose that WIP-YAP/TAZ represent a new pathway that connects mtp53-grotwh factor receptor signals via Akt with oncogenesis. Based on this multistep tumorigenic model, at least in gliomas, it is tantalizing to propose that WIP inhibitors may be applied as an effective anti-cancer therapy.

The pathway regulated by WIP and Akt isoform may control cell viability and proliferation through the control of YAP/TAZ stability in gliomas. Oncogenic mutations in p53 enhances the proliferation and survival signalling throughout, among others, growth factor receptor with tyrosine kinase activity (GFRs), increasing the activity of several pathways such as PI3K-Akt and Ras/Raf/Erk.

TICs derived from gliomas with mutations in p53 correlated with high levels of WIP protein and YAP/TAZ. In these TICs, the presence of high levels of WIP was essential to the proliferation and CSC phenotype via mtp53 and Akt2, maintaining high levels of YAP/TAZ by preventing its degradation in the proteasome.
